# Supplementary material for: Comparison of HPLC, HPTLC, and In Silico Lipophilicity Parameters Determined for 5-Heterocyclic 2-(2,4-Dihydroxyphenyl)-1,3,4-thiadiazoles
Source: Molecules. 2024 May 24;29(11):2478. doi: 10.3390/molecules29112478 (PMC11173846; doi:10.3390/molecules29112478)
Supplement: Supplementary file 1 [file molecules-29-02478-s001.zip › molecules-3015369-supplementary.pdf]

Comparison of HPLC, HPTLC and in silico lipophilicity parameters determined for 5-heterocyclic 2-(2,4-dihydroxyphenyl)-1,3,4-thiadiazoles

Beata Paw, Rafał Śliwa, Łukasz Komsta, Bogdan Senczyna, Monika Karpińska and Joanna Matysiak

Table S1. (-S) and R_{Mw} parameters of Soczewiński-Wachtmeister's equation (Eq. 1) obtained using HPTLC chromatography with the C-18 stationary phase and MeOH or ACN as an organic modifier.

**No.**	**-S (T/C18/ MeOH)**	**R_{Mw} (C18/ MeOH)**	**R^2**	**n**	**-S (T/C18/ ACN)**	**R_{Mw} (C18/ACN)**	**R^2**	**n**
**1.**	4.1491	3.3060	0.9942	5	2.92210	1.7959	0.9890	5
**2.**	3.7446	3.1040	0.9892	5	2.9252	2.0450	0.9825	5
**3.**	6.2529	5.5344	0.9604	5	3.0943	2.4520	0.9849	5
**4.**	5.7549	5.3404	0.9928	5	3.206	2.8047	0.9866	5
**5.**	5.4240	5.3266	0.9827	5	4.3784	3.9912	0.9704	5
**6.**	4.4737	3.6879	0.9855	5	3.3074	2.1534	0.9908	5
**7.**	4.6211	3.9370	0.9775	5	3.4874	2.3350	0.9497	5
**8.**	4.4564	3.9292	0.9952	5	3.4831	2.6342	0.9920	5
**9.**	2.5203	2.5407	0.9738	5	2.0444	1.5988	0.9677	5
**10.**	4.9183	4.1719	0.9629	5	1.7921	1.3930	0.8634	5
**11.**	4.6574	4.1639	0.9614	5	1.9886	1.8413	0.9964	5
**12.**	4.7817	4.4588	0.9896	5	2.2903	2.1437	0.9732	5
**13.**	3.7882	3.1846	0.9506	5	2.1757	1.4946	0.9456	5
**14.**	3.8211	3.3937	0.9827	5	2.1560	1.6266	0.9663	5
**15.**	5.8883	5.5258	0.9950	5	3.6982	3.1371	0.9932	5
**16.**	5.3342	5.3901	0.8919	5	4.1206	3.7963	0.9996	5
**17.**	6.6825	6.3131	0.9942	5	3.9561	3.4444	0.9982	5
**18.**	5.8192	4.8550	0.9803	5	4.0162	2.8388	0.9849	5

Table S2. (-S) and R_{Mw} parameters of Soczewiński-Wachtmeister's equation (Eq. 1) obtained using HPTLC chromatography with the C-18 stationary phase and acetone or dioxane as an organic modifier.

**No.**	**-S (T/C18/ acet)**	**R_{Mw} (C18 /acet)**	**R^2**	**n**	**-S (T/C18/ diox)**	**R_{Mw} (C18/diox)**	**R^2**	**n**
**1.**	4.3796	2.9669	0.9870	5	4.5408	2.6972	0.9928	5
**2.**	4.4803	3.0399	0.9912	5	4.9745	3.0933	0.9956	5
**3.**	5.6259	4.0223	0.9976	5	6.1618	4.0825	0.9984	5
**4.**	5.7954	4.3149	0.9980	5	6.6287	4.5000	0.9970	5
**5.**	6.7547	5.2790	0.9968	5	8.5604	6.1130	0.9876	5
**6.**	4.7855	3.3162	0.9972	5	5.4583	3.4280	0.9902	5
**7.**	4.4976	3.1608	0.9934	5	5.9093	3.8132	0.9964	5
**8.**	5.4443	3.9997	0.9970	5	6.2458	4.1748	0.9976	5
**9.**	3.5181	2.2885	0.9908	5	3.8198	2.1989	0.9988	5
**10.**	4.7123	3.2671	0.9876	5	5.7045	3.5522	0.9637	5
**11.**	4.6921	3.2164	0.9880	5	4.8941	2.9864	0.9857	5
**12.**	4.1765	3.0544	0.9924	5	5.7639	3.6677	0.9900	5
**13.**	4.7694	3.2632	0.9590	5	4.8418	2.8815	0.9567	5
**14.**	3.8456	2.5873	0.9934	5	4.7836	2.8799	0.9884	5
**15.**	6.8665	5.2117	0.9841	5	7.8364	5.4621	0.9892	5
**16.**	6.8274	5.2793	0.9942	5	8.6995	6.1654	0.9843	5
**17.**	7.0519	5.6072	0.9894	5	8.1574	5.9482	0.9934	5
**18.**	6.0199	4.4378	0.9968	5	6.7770	4.5293	0.9876	5

Table S3. (-S) and R_{Mw} parameters of Soczewiński-Wachtmeister's equation (Eq. 1) obtained using HPTLC chromatography with the C-8 stationary phase and MeOH or ACN as an organic modifier.

**No.**	**-S (T/C8/ MeOH)**	**R_{Mw} (C8/MeOH)**	**R^2**	**n**	**-S (T/C8 /ACN)**	**R_{Mw} (C8 /ACN)**	**R^2**	**n**
**1.**	4.1315	3.0797	0.9916		3.8243	2.1835	0.9978	5
**2.**	4.9429	3.7959	0.9982		3.6492	2.2625	0.9984	5
**3.**	6.0444	4.8463	0.9868		4.4172	2.8578	0.9964	5
**4.**	5.9838	4.9041	0.9960		4.5680	3.0813	0.9934	5
**5.**	6.5440	5.5285	0.9970		5.2285	3.7721	0.9954	5
**6.**	4.3476	3.2212	0.9946		4.2628	2.4555	0.9916	5
**7.**	4.4356	3.2956	0.9918		4.6640	2.6188	0.9860	5
**8.**	5.0126	3.9643	0.9948		4.3250	2.7835	0.9866	5
**9.**	2.8400	2.2257	0.9239		3.5004	2.2232	0.9851	5
**10.**	4.2310	3.4404	0.9655		2.7574	1.7456	0.9411	5
**11.**	4.0306	3.5402	0.9894		2.6664	2.1297	0.9948	5
**12.**	4.2900	3.8391	0.9952		2.9978	2.4195	0.9914	5
**13.**	4.3787	3.3996	0.9767		3.2117	1.8695	0.9894	5
**14.**	4.3890	3.5117	0.9964		4.1175	2.5614	0.9657	5
**15.**	6.2773	5.3333	0.9479		5.5175	3.8605	0.9855	5
**16.**	7.5941	6.5014	0.9946		6.6108	4.7899	0.9712	5
**17.**	6.1492	5.2990	0.9479		5.8419	4.1478	0.9781	5
**18.**	6.0795	4.6757	0.9996		4.9035	3.1100	0.9938	5

Table S4. (-S) and R_{Mw} parameters of Soczewiński-Wachtmeister's equation (Eq. 1) obtained using HPTLC chromatography with the C-8 stationary phase and acetone or dioxane as an organic modifier.

**No.**	**-S (T/C8 /acet)**	**R_{Mw} (C8/acet)**	**R^2**	**n**	**-S (T/C8 /diox)**	**R_{Mw} (C8 /diox)**	**R^2**	n
**1.**	3.7811	2.4959	0.9900	5	4.1866	2.3982	0.9839	5
**2.**	4.1706	2.7394	0.9831	5	4.9249	2.9717	0.9930	5
**3.**	5.4097	3.6387	0.9777	5	6.2724	4.0058	0.9970	5
**4.**	5.7869	3.9813	0.9730	5	6.2868	4.0984	0.9994	5
**5.**	7.0061	5.0093	0.9853	5	7.5512	5.1706	0.9920	5
**6.**	4.5325	2.9312	0.9896	5	5.1703	3.1046	0.9966	5
**7.**	4.8239	3.1183	0.9769	5	5.3699	3.3106	0.9898	5
**8.**	5.3519	3.6573	0.9742	5	6.1010	3.9029	1.0000	5
**9.**	3.5777	2.1722	0.9692	5	5.2716	3.0424	0.9539	5
**10.**	4.9708	3.2475	0.9910	5	5.3472	3.1758	0.9938	5
**11.**	3.8896	2.6908	0.9801	5	4.9161	3.0005	0.9952	5
**12.**	4.9854	3.5097	0.9700	5	4.8227	3.0526	0.9934	5
**13.**	3.6842	2.2444	0.9944	5	3.5098	1.8586	1.0000	5
**14.**	4.4808	2.8818	0.9906	5	4.9916	2.8806	0.9944	5
**15.**	7.0681	5.0068	0.9803	5	7.6906	5.1527	0.9988	5
**16.**	8.0992	5.9450	0.9821	5	9.2740	6.4537	0.9467	5
**17.**	7.3211	5.2993	0.9946	5	8.0259	5.4738	0.9994	5
**18.**	5.6323	3.9973	0.9948	5	8.8171	5.4468	0.9896	5

Table S5. (-S) and log k_w parameters of Soczewiński-Wachtmeister's equation (Eq. 2) obtained using HPLC chromatography with the C-8 stationary phase and MeOH or ACN as an organic modifier.

No.	-S (C/ C8/MeOH)	log k_w (C8/MeOH)	R^2	n	-S (C/ C8/ACN)	log k_w (C8/ACN)	R^2	n
**1.**	6.0082	4.2043	0.9816	5	3.4819	1.8748	0.9962	11
**2.**	6.1209	4.5676	0.9954	5	3.1457	2.0112	0.9866	10
**3.**	6.6477	5.3800	0.9772	5	3.3024	2.3327	0.9934	10
**4.**	6.9580	5.7912	0.987	5	3.4306	2.6173	0.9913	9
**5.**	6.9674	6.1111	0.9944	4	3.6063	3.0550	0.9886	7
**6.**	5.5916	4.1185	0.9898	7	3.7404	2.2108	0.9937	11
**7.**	5.8849	4.4413	0.9617	6	3.9235	2.3706	0.9934	9
**8.**	5.9408	4.7382	0.9967	5	3.4789	2.4338	0.9967	9
**9**	5.6396	3.9890	0.9992		3.6442	2.0671	0.9885	
**10.**	5.7388	4.2394	0.9812		4.2741	2.3100	0.9907	9
**11.**	5.3577	4.1136	0.9878	5	2.9047	1.6312	0.9800	10
**12.**	6.4501	5.0221	0.9971	6	2.9388	1.8312	0.9657	9
**13.**	5.2564	3.5488	0.9827	5	3.0501	1.4122	0.984	9
**14.**	6.4211	4.5421	0.9833	5	3.3654	1.6737	0.9899	9
**15.**	7.2800	6.1950	0.9984	5	3.7454	2.9768	0.9934	
**16.**	7.4969	6.5330	0.9947	5	3.9605	3.**3937**	0.9855	6
**17.**	7.6416	6.5561	0.9945	5	3.8024	3.0725	0.9846	7
**18.**	8.0456	6.1282	0.9967	5	3.6822	2.4879	0.9945	10

Table S6. (-S) and log k_w parameters of Soczewiński-Wachtmeister's equation (Eq. 2) obtained using HPLC chromatography with C-18 and IAM stationary phases.

**No.**	**-S (C/C18/MeOH)**	**log k_w (C18/MeOH)**	**R^2**	**n**	**-S (IAM)**	**log k_w (IAM)**	**R^2**	**n**
**1.**	4.3570	2.7375	0.9996	5	6.5961	1.871	0.9890	9
**2.**	4.2329	2.9928	0.9947	5	4.8737	2.2701	0.9775	6
**3.**	4.9383	4.1197	0.9978	5	4.9043	2.8582	0.9866	5
**4.**	5.5585	4.9777	0.9985	5	5.9078	3.2923	0.9697	5
**5.**	6.3647	5.8533	0.9989	5	5.6231	3.5149	0.9959	4
**6.**	4.21977	3.0278	0.9989	5	4.7601	1.9734	0.9856	8
**7.**	5.2481	3.8762	0.9999	5	6.0858	2.1166	0.9928	8
**8.**	5.3917	4.2955	0.9999	5	5.5556	2.7874	0.9799	7
**9**	3.8405	2.9331	0.9978	5	5.1757	1.2283	0.9983	8
**10.**	4.4841	4.1724	0.9996	5	4.7935	2.6233	0.9868	6
**11.**	4.2065	3.3785	0.9978	4	4.7404	2.0075	0.9750	8
**12.**	4.2984	3.4873	0.9983	4	7.2573	2.0537	0.9968	9
**13.**	3.6556	2.5789	0.9999	4	4.9953	1.9665	0.9653	8
**14.**	4.0965	3.0298	0.9966	5	7.8465	2.1734	0.983	9
**15.**	5.9087	5.0220	0.9968	5	6.1233	3.5084	0.9943	5
**16.**	6.3092	*5.6898*	0.9965	5	6.3109	3.8012	0.9895	4
**17.**	6.2696	5.3245	0.9972	5	7.1295	3.6028	0.9973	5
**18.**	6.5546	4.6912	0.9974	5	6.5851	2.5350	0.9859	6

Table S7. (-S) and log k_w parameters of Soczewiński-Wachtmeister's equation (Eq. 2) obtained using HPLC chromatography with the Chol stationary phase.

No.	-S (Chol/MeOH)	log k_w (Chol/MeOH)	R^2	n
**1.**	4.3316	3.1313	0.9749	8
**2.**	5.0425	3.8947	0.9815	8
**3.**	5.0868	4.8208	0.9744	5
**4.**	5.3300	5.2230	0.9622	4
**5.**	6.1532	6.1235	0.9639	4
**6.**	4.5604	3.7689	0.9909	8
**7.**	4.7103	3.9254	0.9929	8
**8.**	5.3923	4.7107	0.9832	6
**9.**	4.0111	4.1940	0.9892	4
**10.**	4.8072	4.1155	0.9679	6
**11.**	4.0012	3.5685	0.9771	7
**12.**	4.117	3.6624	0.9761	7
**13.**	3.9221	3.1856	0.9783	8
**14.**	3.9588	3.2877	0.9812	8
**15.**	6.2661	5.7619	0.9587	4
**16.**	6.2481	5.9081	0.9820	4
**17.**	6.5357	5.9940	0.9579	4
**18.**	5.7959	4.4579	0.9865	8
